# When Work Integrated Learning Costs Too Much: The Hidden Toll of Clinical Placements

**DOI:** 10.1002/jmrs.70032

**Published:** 2025-11-11

**Authors:** Vicki Braithwaite, Therese Gunn, Pamela Rowntree, Judith Singleton

**Affiliations:** ^1^ Discipline of Medical Radiation Sciences, Faculty of Health, School of Clinical Sciences Gardens Point Campus Brisbane Queensland Australia; ^2^ Faculty of Health, School of Clinical Sciences Gardens Point Campus Brisbane Queensland Australia; ^3^ Pharmacy Discipline, Faculty of Health, School of Clinical Sciences Gardens Point Campus Brisbane Queensland Australia

**Keywords:** clinical supervision, diagnostic imaging, education, practicum, radiography

## Abstract

**Introduction:**

Work Integrated Learning (WIL) experiences are a key component in medical imaging (MI) studies. However, these unpaid placements often result in significant financial stress, otherwise known as ‘placement poverty’, due to reduced income during placement and associated costs. Despite significant research in other allied health disciplines, the impact on MI students remains under‐researched.

**Methods:**

A structured survey based upon the WIDE survey originally developed in New Zealand was administered by the University's Qualtrics platform. Ethics approval was granted (Ethics # 6894). The survey targeted students in their final 2 years of the four‐year Honours degree, along with students within 2 years post‐graduation. The survey collected both quantitative and qualitative data on financial stress during WIL placements. Of 250 eligible participants, 56 responded, with 28 complete responses included in the analysis.

**Results:**

Findings have demonstrated MI students experience substantial financial hardship during WIL placements. Key stressors included travel, accommodation, uniforms, and increased daily living costs. Full‐time placement schedules limited students' ability to maintain part‐time employment, with rural placements further increasing their financial burden. Students reported impacts on mental health, academic performance, and overall wellbeing. Financial stress led some students to reduce their study load, take leave from the course or consider course withdrawal.

**Conclusion:**

This study highlights the significant financial challenges facing MI students during WIL placements, with implications for students' retention and workforce sustainability. Targeted financial support, flexible placement models, and policy reform are urgently needed to ensure equitable training and to address the ongoing workforce shortage.

## Graphical abstract

Medical imaging students face major financial stress during unpaid clinical placements, with costs for travel, accommodation, and lost income impacting mental health and study success. This stress can affect their mental health, studies, and even lead some to consider leaving their course. Urgent action is needed to provide support and ensure workforce sustainability.

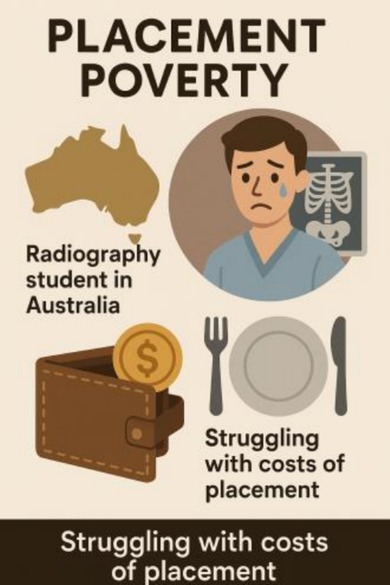

## 1 Introduction

For students in medical radiation sciences, Work Integrated Learning (WIL) placements are not optional; they are a mandatory requirement of the curriculum and a prerequisite for professional registration with the Australian Health Practitioner Regulation Agency (Ahpra). WIL placements are designed to immerse students in the clinical setting, where they can observe, participate, and gradually assume greater responsibility under the supervision of qualified practitioners [1]. This experiential learning is vital for developing technical skills, clinical reasoning, communication, and professional behaviour [1]. Despite their educational value, WIL placements present significant challenges, particularly in terms of financial stress, a term referred to as *placement poverty* [2, 3, 4]. Placement poverty refers to the financial stress experienced by students during their unpaid WIL placements, often due to reduced income from employment and increased costs associated with travel, accommodation, and living expenses [2, 3, 4].

The financial impact of unpaid WIL placements on students in allied health, medicine, social work, and nursing disciplines in Australia is well‐documented in research [2, 3, 5, 6, 7, 8, 9]. Over recent years, research has consistently highlighted the need for enhanced financial support for students undertaking these placements [2, 3, 4]. WIL placements are a cornerstone of education and professional development for allied health students, including those pursuing degrees in medical radiation sciences. These placements serve as a bridge between academic learning and professional practice, offering students the opportunity to apply theoretical knowledge in real‐world healthcare environments. Ahpra outlines the importance of WIL placements, stating that they enable students to consolidate their learning and develop the practical competencies required for safe and effective practice [1].

Whilst WIL placement is an integral component of Allied Health courses in Australia, and although support for medical imaging (MI) students has been studied previously as part of wider research into Allied health, there has been a dearth of research into the impact of financial stress carried by medical imaging students [4, 5, 6, 7, 8, 9]. At Queensland University of Technology (QUT), MI students undertake approximately 1920 h (equivalent to 52 weeks) of WIL placement over the 4 years of the degree, with the next closest in terms of WIL hours being Radiation Therapy (RT) with 1462 h, and then Podiatry with 940 h over their four‐year degrees (S Cameron, QUT Work Integrated Learning Support, email, March 12, 2025). Research into Nurse education debt, as reported by the National Centre for Social and Economic Modelling (NATSEM), describes this generation as the educated poor [8]. In a 2019 study by Grant‐Smith, they found that WIL placements have a significant financial impact on students who are financially independent, most notably among those who rely on paid employment as their income source [9]. This income source is impacted by their WIL placement, with students either forgoing work or attempting to manage both work and their WIL placement, leading to fatigue and decreased wellbeing. For Australian medical radiation sciences students, the move from a three‐year undergraduate program with a paid supported practicum placement (SPP) graduate year to a four‐year full‐time undergraduate program in the last 10 years has further increased their financial debt. Furthermore, the increased year of WIL placement has added to the growing financial stress on these students.

Multiple Australian‐based studies on the impact of WIL on student wellbeing in Allied Health training have reported that there are considerable levels of financial stress on students [4, 7, 9]. WIL is noted as the primary cause due to the loss or reduction of employment while undertaking the unpaid WIL placement‐related component of their study. Only one study researched medical imaging students within allied health student participants, with less than 7.5% (41/552) of the respondents enrolled in an MI degree [9]. There is no other research investigating specifically the impact of WIL placement on MI students.

By highlighting the issue of WIL placement‐related financial hardship, this study seeks to advance the wider discussion of this financial stress in medical radiation sciences education. Recognizing and addressing the financial challenges faced by students is essential for the medical radiation sciences profession to meaningfully support the development of future practitioners. While the findings align with existing literature indicating that students who undertake WIL placements experience financial disadvantage, this study is the first to specifically document the experiences of medical imaging students alone, offering a focused contribution to the field.

## 2 Methods

### 2.1 Survey Design and Distribution

A structured survey, adapted, with permission, from the WIDE survey originally developed in New Zealand and previously administered to medical students, was deployed using QUT's Qualtrics platform [10]. Following ethics approval, the survey was distributed to Queensland University of Technology (QUT) Bachelor of Medical Imaging (BMI(Hons)) third‐ and fourth‐year MI students, as well as recent graduates (within 2 years of course completion). This cohort was selected due to their direct experience with the course's extensive WIL requirements, which span over 35 weeks of supervised clinical placement in their final 2 years. The survey was designed to collect both quantitative and qualitative data to explore student debt on MI students' lives while undertaking their MI qualification, including experiences related to their WIL placements. The questionnaire included a mix of closed‐ended questions to gather quantitative data and open‐ended questions to capture students' personal insights and lived experiences. Ethics approval was granted by the Queensland University of Technology Human Ethics Committee #6894.

### 2.2 Participants

Participants were recruited via email, which included the participant information sheet and a link to the survey. A total of 56 responses (*n* = 56/250; response rate of 22.4%) were collected from 250 possible participants. Out of the 56 responses received, only 28 of the 56 surveys completed were deemed suitable for analysis, resulting in a 50% exclusion rate (*n* = 28/56). The primary reason for rejection was incomplete survey data, with numerous unanswered questions, particularly those related to student debt.

### 2.3 Data Collection

The survey was open from May to September 2024. Three email requests were sent in May, July, and September 2024.

## 3 Results

Due to the low response rate (*n* = 28), inferential statistical analyses (chi‐squared tests) were unable to be performed as not all of the test assumptions were met. Therefore, the results are presented using descriptive statistics. The qualitative data referenced in this article were collected through open‐ended questions embedded within the Qualtrics survey. These questions were designed to elicit participants' personal views and perceptions related to their work/study/life balance and any impact on their mental and physical health. Responses were submitted anonymously. Responses have been collected; however, data analysis of these responses has not been included at this time as qualitative data collection (interview) is ongoing.

**FIGURE 1:**
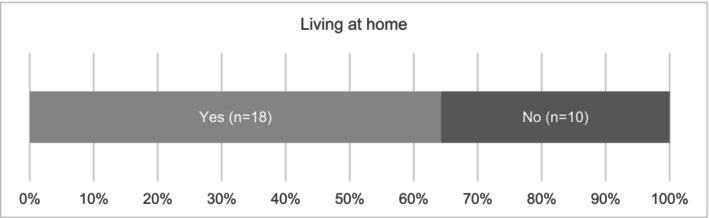
Students living at home during their study.

**FIGURE 2:**
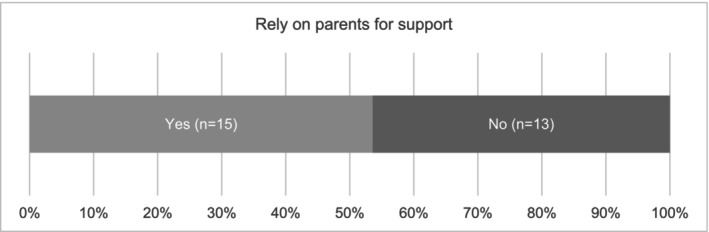
Students relying on parents for financial support during study.

Of those participants responding to the Qualtrics survey, 64% (18/28) lived at their parents' home whilst studying (Figure 1), with just over half (15/28) relying on their parents for ongoing financial support to complete their studies (Figure 2), which suggests nearly half are trying to maintain self‐support and independence whilst studying.

Participants were requested to evaluate the extent to which any paid employment undertaken during their studies had affected their academic performance. Nearly half of the participants reported that their work commitments had impacted their studies to some degree.

Subsequently, participants were asked whether their employment had influenced their WIL placements. Over half of the participants indicated that their job responsibilities had a noticeable impact on their placement experience, while fewer than 15% reported no effect.

**FIGURE 3:**
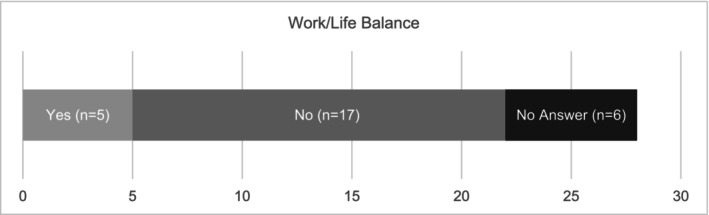
Students focus on work/life balance.

Students were also asked if they felt they had a good work/study/life balance. Concerningly, 22% (6/28) of participants chose not to respond to the question, an omission that may itself reflect underlying stress or discomfort. Among those who did respond, nearly 80% (17/22) reported *not* having a satisfactory work‐life balance, while fewer than 23% (5/22) felt they did. (See Figure 3).

**FIGURE 4:**
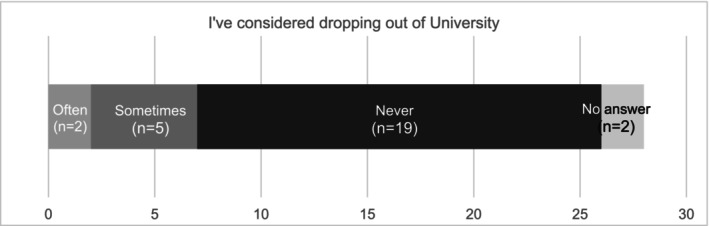
Student attrition risk: Consideration of dropping out of the course.

Participants were then asked if their financial situation is a cause of worry/stress. Close to half the participants indicated they were already experiencing financial stress. Participants were asked how often they felt worried/stressed about the implications of their student debt. As demonstrated in Figure 4, one‐quarter (25%; 7/28) of survey participants reported considering withdrawing from university and subsequently from the medical imaging profession to alleviate their debt pressures. These students are facing the difficult challenge of balancing the need to earn an income with the demands of their academic studies.

Participants were also invited to describe the impact of their WIL placements on their financial stress. One participant shared that persistent financial concerns had significantly affected their mental health, stating that it made studying more stressful and led to constant anxiety about spending.

I continually worry about my finances, and this has had an effect on my mental health. It has made studying stressful and makes me always think about how I am spending my money. (P7)

Another participant described the experience as ‘exhausting,’ noting the difficulty of balancing academic demands with the need to work in order to cover living expenses, transportation, and future goals. The added burden of loan indexation was cited as a further source of stress.

Its exhausting. You are focussing on studying to get your degree but at the same time you're trying to work to help pay bills, socialise, placement (transport) and future goals (car). Furthermore, when the indexation was applied, it made it a lot more stressful. (P28)

A particularly detailed response from one participant illustrated how financial stress not only limited time available for academic pursuits but also negatively influenced basic wellbeing. The student reported working nearly full‐time to afford rent and living costs, which in turn reduced their capacity to study effectively. They also noted that financial constraints led to poor nutrition, with regular, balanced meals becoming a rarity.

Whilst I was studying, financial stress would often impact my psychological well‐being in a way that would further bleed into stress regarding study and academic achievement. The requirement for me to work almost full time to support the general costs of living and rent whilst studying meant that there was less time for me to spend on actively studying or my academic pursuits. Secondary impacts of financial stress meant that nutrition was of little import, with regular and nutritionally sound meals being infrequent. (P10)

## 4 Discussion

Medical imaging is a vital component of contemporary healthcare, integral to accurate diagnosis and the informed guidance of clinical decision‐making across a patient's diagnostic journey. Positioned within multidisciplinary teams, medical imaging offers insights into patient health and wellbeing and contributes meaningfully to the delivery of patient‐centre care. By supporting timely and precise diagnostic procedures, medical imaging ensures that diagnostic pathways are responsive to individual patient needs and aligned with best practice.

A reduction in the number of qualified radiographers poses a serious risk to the quality and accessibility of healthcare services nationwide [11]. However, the pathway to this qualification is increasingly constrained by financial barriers. The cumulative impact of these financial pressures can significantly affect students' financial wellbeing and subsequently their physical health, mental well‐being, and academic performance [7, 9, 12].

The study has highlighted, within the student reflections, the broader implications of financial hardship on student wellbeing and academic success, particularly during their WIL placement periods. MI students face substantial financial stress due to the costs associated with WIL placements. These costs include travel, accommodation, uniforms, and equipment (such as sidemarkers and radiation badges) [2]. Unlike traditional academic semesters, WIL placements often require students to commit to full‐time hours, typically 40 h per week, and in many instances within the 24‐h site roster requirements in clinical settings. This leaves little to no time for part‐time employment, resulting in loss of income and increased financial stress. At QUT, students undertake 52 weeks of placement across their 4‐year course, significantly more than other allied health degrees. These placements are required across a variety of clinical sites, including mandatory rural or regional placements, which further increase costs.

Many students rely on part‐time employment to support themselves financially during their studies [4, 5, 6, 7, 9, 12]. However, when students are undertaking full‐time WIL placements, they are often forced to leave their part‐time jobs, leading to significant financial stress [9]. The financial demands of WIL placements are multifaceted. Rural and regional placements often necessitate temporary relocation, adding to accommodation and living expenses. Daily costs such as meals and parking further intensify the financial burden. For students already managing tuition fees, textbooks, and other academic expenses, these additional costs can be overwhelming and further compound the financial pressure. Financial barriers during education contribute to higher dropout rates, limiting the pipeline of new graduates and placing additional strain on existing staff [13].

This has implications for the profession going forward as the medical radiation sciences profession is experiencing a workforce shortage [14]. The Australian Government's Occupation Shortage List confirms that diagnostic radiographers are in shortage across most states and territories [14]. Although the Medical Radiation Practice Board of Australia (MRPBA) reports a modest annual increase in radiographers annually, this growth is insufficient to meet rising demand. Recent data indicates the number of medical imaging graduates is only at status quo levels, rather than increasing as required; a statistic that should raise concern among educators, policymakers, and healthcare leaders alike [14]. Health Workforce Australia notes the need for an increased workforce to support the growing health needs across the population; however, current graduate numbers are not meeting the workforce needs. This study indicates that the lack of growth in graduate numbers is not merely a reflection of academic performance or interest in the field of medical radiation sciences but could be a symptom of systemic barriers that hinder student retention and progression. From this study's findings, financial hardship, lack of support during placements, and limited flexibility in current education models all appear to be contributing factors.

Lack of financial support can negatively affect students' mental health, contributing to anxiety, depression, chronic stress, and burnout, which can also impact academic performance [12]. This financial stress can have far‐reaching consequences. Some students may be forced to make difficult choices, such as reducing their study load, incurring additional debt, or withdrawing from their course [9, 12]. Financial stress can also limit students' ability to fully engage in their WIL placements [2]. Grant‐Smith et al. have demonstrated the ongoing impact of WIL placements on student wellbeing [7, 9]. This empirical research was across several degree offerings at QUT in Health and Social Services, Education, and Nursing, all with WIL placements as part of their undergraduate programs. Findings showed that while students could see the learning opportunities offered in their WIL placements required as part of the course, many found WIL placements impacted particularly in terms of financial stress and work‐life balance [7, 9, 11].

Students from lower socioeconomic backgrounds, rural areas, or those without family financial support are disproportionately affected, exacerbating existing inequalities and reducing diversity within the profession [9, 11]. These students may struggle more to complete their education and enter the profession, reducing diversity within the field [3]. The implications of this decline are far‐reaching. With fewer graduates than required entering the workforce, existing staff face increased workloads, which can lead to burnout and further attrition. This cycle places additional strain on healthcare systems already grappling with rising patient demand and complex diagnostic needs [8]. Moreover, rural and remote communities, already underserved in terms of healthcare access, are likely to be disproportionately affected by workforce shortages in medical imaging [8].

Addressing student financial stress is crucial to ensure equitable access to training and to sustain the future workforce. This involves providing financial support, improving access to affordable accommodation, and offering flexible placement options to accommodate students' needs [7, 11]. The Commonwealth Practicum Payment (CPP), introduced in July 2025, provides $319.50 per week to eligible students in teaching, midwifery, and social work [15]. Extending this support to medical radiation sciences students could alleviate placement‐related financial stress. Additionally, the 20% reduction in student loan debt and revised payment thresholds from June 2025 offer further relief [15]. However, existing scholarships remain limited and often inaccessible, underscoring the need for broader systemic reform. By acknowledging and addressing the interconnected challenges of placement poverty, rising education costs, and workforce sustainability, the Australian medical imaging profession can take proactive steps to secure its future. Universities offer a range of student support services, but increasing demand has strained accessibility. In response, some medical imaging departments have acted of their own volition, establishing informal food banks and care package stations to provide immediate relief to students in need. Australia's peak professional body, Australian Society of Medical Imaging and Radiation Therapy (ASMIRT), is to be commended for its innovative approach to student support, with the introduction of the $500 student placement support grant [16]. This grant is available to ASMIRT medical radiation sciences student members undertaking a WIL Placement in their final year of study. This grant is additional to the rural clinical placement scheme offered by ASMIRT for students attending a WIL placement in a rural placement for required travel and accommodation [17].

A limitation of this study was the very low response rate; regardless, the responses (and particularly the qualitative data) raise important discussion points for the profession. WIL placements are an essential part of medical imaging education, providing students with essential clinical experience and professional development. However, these placements present a range of challenges that can significantly affect students' academic performance, physical health, and overall well‐being. Among the most pressing concerns is financial stress, an issue that demands urgent and coordinated attention from all stakeholders in medical radiation sciences education.

University institutions and the medical imaging profession should investigate whether it is possible for flexible placement schedules to accommodate part‐time work by students. Currently, our training models require a 40‐h WIL working week over a 24‐h period where applicable and completion of the course within 4 years. Can this be reimagined for some students? Analysis is required on how this will impact graduates and our current workforce needs. The current situation may be limiting the potential intake of students, limiting it to those who have parental financial support and excluding some from lower socio‐economic areas. With students looking to reduce their study load with part‐time study or alternatively take study leave, an option for flexibility in placement scheduling may offer another avenue for support, potentially allowing students to maintain part‐time employment. However, any shift in educational training models must be carefully evaluated to ensure it does not compromise graduate readiness or workforce capacity. The current requirement of a 40‐h WIL week, often across a 24/7 roster, reflects the realities of clinical practice and must be weighed against the need for student sustainability. Supporting students financially, academically, and emotionally is not only an investment in their individual success but also in the resilience and quality of Australia's healthcare system. Ensuring that students are well‐prepared and well‐supported will help cultivate a generation of competent, compassionate, and capable medical imaging professionals ready to meet the evolving needs of the community.

## 5 Conclusion

Addressing the challenges identified in this study requires a multifaceted and collaborative approach. Universities, professional bodies, and government agencies must work together to implement sustainable solutions that support students throughout their educational journey. This includes expanding financial assistance programs, increasing access to targeted scholarships, and exploring innovative placement models that reduce financial and logistical burdens. Placement poverty, where students face significant financial stresses during the mandatory clinical placements, can hinder their progression and completion. This not only exacerbates current workforce shortages but also compromises the medical radiation sciences profession's capacity to maintain a workforce. Additionally, workforce planning strategies must prioritise the recruitment and retention of new graduates to ensure a stable and sustainable future for the profession.

## Ethics Statement

Ethics approval was granted by the Queensland University of Technology (QUT) Human Ethics Committee #6894.

## Conflicts of Interest

The authors declare no conflicts of interest.

## Data Availability

The data that support the findings of this study are available from the corresponding author upon reasonable request.
